# The Easy-to-Use SARS-CoV-2 Assembler for Genome Sequencing: Development Study

**DOI:** 10.2196/31536

**Published:** 2022-03-14

**Authors:** Martina Rueca, Emanuela Giombini, Francesco Messina, Barbara Bartolini, Antonino Di Caro, Maria Rosaria Capobianchi, Cesare EM Gruber

**Affiliations:** 1 Laboratory of Virology and Biosafety Laboratories National Institute for Infectious Diseases “Lazzaro Spallanzani” Istituto di Ricovero e Cura a Carattere Scientifico Rome Italy; 2 Laboratory of Microbiology and Biological Bank National Institute for Infectious Diseases “Lazzaro Spallanzani” Istituto di Ricovero e Cura a Carattere Scientifico Rome Italy; 3 UniCamillus - Saint Camillus International University of Health Sciences Roma Italy

**Keywords:** SARS-CoV-2 genome, bioinformatics tool, NGS data analysis, COVID-19, genome, health informatics, bioinformatic, digital tools, algorithms

## Abstract

**Background:**

Early sequencing and quick analysis of the SARS-CoV-2 genome have contributed to the understanding of the dynamics of COVID-19 epidemics and in designing countermeasures at a global level.

**Objective:**

Amplicon-based next-generation sequencing (NGS) methods are widely used to sequence the SARS-CoV-2 genome and to identify novel variants that are emerging in rapid succession as well as harboring multiple deletions and amino acid–changing mutations.

**Methods:**

To facilitate the analysis of NGS sequencing data obtained from amplicon-based sequencing methods, here, we propose an easy-to-use SARS-CoV-2 genome assembler: the Easy-to-use SARS-CoV-2 Assembler (ESCA) pipeline.

**Results:**

Our results have shown that ESCA could perform high-quality genome assembly from Ion Torrent and Illumina raw data and help the user in easily correct low-coverage regions. Moreover, ESCA includes the possibility of comparing assembled genomes of multisample runs through an easy table format.

**Conclusions:**

In conclusion, ESCA automatically furnished a variant table output file, fundamental to rapidly recognizing variants of interest. Our pipeline could be a useful method for obtaining a complete, rapid, and accurate analysis even with minimal knowledge in bioinformatics.

## Introduction

Next-generation sequencing (NGS) has reached a pivotal role in the field of emerging infectious diseases by enhancing the development capacity of new diagnostic methods, vaccines, and drugs [1,2]. Moreover, a key role has been recognized for sequence data production and sharing in outbreak response and management [3-5]. In the current COVID-19 epidemic, more than 6 million full genome sequences of SARS-CoV-2 have been deposited in publicly accessible databases in the arc of 1 year (ie, GISAID) [6,7]. SARS-CoV-2 genome surveillance on a global scale is permitting real-time analysis of the outbreak, with a direct impact on the public health response. This contribution includes the tracing of SARS-CoV-2 spread over time and space, evidence of emerging variants that may affect pathogenicity, transmission capacity, diagnostic methods, therapeutics, or vaccines [8-11]. Recently divergent SARS-CoV-2 variants are emerging in rapid succession, harboring multiple deletions and amino acid mutations. Some mutations occur in the receptor-binding domain of the spike protein and are associated with an increase of angiotensin-converting enzyme 2 (ACE2) affinity as well as a potential reduction of polyclonal human plasma antibody efficacy [12,13]. The growing contribution of sequence information to public health is driving global investment in sequencing facilities and scientific programs [14,15]. The falling cost of generating genomic NGS data provides new chances for sequencing capacity expansion; however, many laboratories have low sequencing capacity and even a lack of expertise for data elaboration.

While sequencing runs can be performed without consolidated experience in the infectious disease field, virus genomic sequence assembly is often a demanding task. Translating SARS-CoV-2 raw read data into reliable and informative results is complex and requires solid bioinformatics knowledge, particularly for low-coverage samples. Some steps can lead to incorrect variant calling and produce erroneous assembled sequences.

Supervision of the sequence assembly to avoid inconsistent or misleading assignment of a virus to a taxonomic lineage or clade [9,10] as well as evaluation of low-coverage samples to prevent loss of epidemiological information are mandatory.

Many tools have been developed to support whole genome sequence reconstruction, starting with reads produced by different NGS platforms. However, most tools have been designed for genome assembly of other viruses and often are able to elaborate only a specific type of data. Some of these tools, for example, have implemented the assembly method for one specific platform (ie, Loretta for PacBio data) [16] or for a specific sequencing approach (ie, UNAGI for Nanopore and Illumina data) [17]. Some sequencing platform manufacturers have proposed pipelines for SARS-CoV-2 genome reconstruction that have been designed to obtain the most accurate sequence from one specific technological output. For example, Illumina developed the DRAGEN tool for SARS-CoV-2 genome analysis, a commercial tool that is temporarily free and available online, while Ion Torrent suggests the iterative refinement meta-assembler (IRMA) for SARS-CoV-2 data analyses, an open-source program developed by the Centers for Disease Control and Prevention (CDC) [18].

We propose the Easy-to-use SARS-CoV-2 Assembler (ESCA) pipeline: a novel reference-based genome assembly pipeline specifically designed for SARS-CoV-2 data analysis. This pipeline was created to support laboratories with limited experience in bioinformatics for SARS-CoV-2 analysis. ESCA can be easily installed and runs in most Linux environments.

## Methods

### Overview

The ESCA pipeline is a reference-based assembly algorithm written for Linux environments and requires only raw reads as input files, without any other information. Two versions of the software are available: one for Illumina paired-end reads in the “fastq.gz” file format and the other for Ion Torrent reads in the “ubam” file format.

The software is designed to process several samples in a single run. All reads (paired or unpaired) must be copied into the same working directory, and then, the program is launched through the command line by typing “StartEasyTorrent” for IonTorrent input or “StartEasyIllumina” for Illumina input. The pipeline than performs all the other passages automatically, as described in the following paragraphs.

The program processes all input reads, dividing them into different samples using file names as identifiers. Illumina paired-end reads are expected to be divided into 2 files that contain “R1” or “R2” to distinguish forward reads from reverse reads.

Sample preprocessing is performed by filtering out all reads with a mean Phred quality score lower than 20 and that are less than 30 nucleotides long.

Filtered reads are mapped on the SARS-CoV-2 reference genome Wuhan-Hu-1 (GenBank Accession Number NC_045512.2) with bwa-mem software [15]; all reads that do not map on the reference genome are then discarded.

Genome coverage is then analyzed: The read-mapping file is converted into “sorted-bam” and “mpileup” files using samtools software [19], and these data are translated into a detailed coverage table that reports the count of nucleotides observed at each position.

The consensus sequence is then reconstructed on the basis of 3 parameters: (1) frequency of nucleotides observed at each position, (2) nucleotide coverage, (3) reference genome sequence.

Briefly, sample parameters for consensus sequence reconstruction are designed to call the nucleotide observed with >50% frequency and with a coverage of >50 reads, but the minimum coverage is reduced at >10 reads if the most frequent nucleotide observed is identical to the nucleotide observed in the reference genome.

For all positions where these parameters are not satisfied, the ESCA pipeline is designed to call “N” to indicate a low coverage position or an intrasample nucleotide variant.

After whole genome reconstruction of all samples, the consensus sequences are aligned with the Wuhan-Hu-1 reference genome using MAFFT software [20], and a mutation table is generated, reporting nucleotide mutations of all the genomes assembled.

### Illumina Data

To test the efficiency of the ESCA pipeline, 228 SARS-CoV-2–positive samples were sequenced with Illumina platforms using the Ion AmpliSeq SARS-CoV-2 Research Panel following the manufacturer’s instructions (ThermoFisher, Waltham, MA). For Illumina samples, whole SARS-CoV-2 genome sequences were assembled using both ESCA and DRAGEN RNA Pathogen Detection v.3.5.15 (BaseSpace) with default parameters.

### Ion Torrent Data

A resequencing assay on Ion Torrent platforms was carried out for the same 228 SARS-CoV-2–positive samples using the Ion AmpliSeq SARS-CoV-2 Research Panel following the manufacturer’s instructions (ThermoFisher).

For Ion Torrent samples, whole SARS-CoV-2 genome sequences were assembled with ESCA and IRMA software [18] using the setting parameters indicated by ThermoFisher, in order to test the consistency of ESCA and IRMA outputs.

### Performance Test

**Figure 1:**
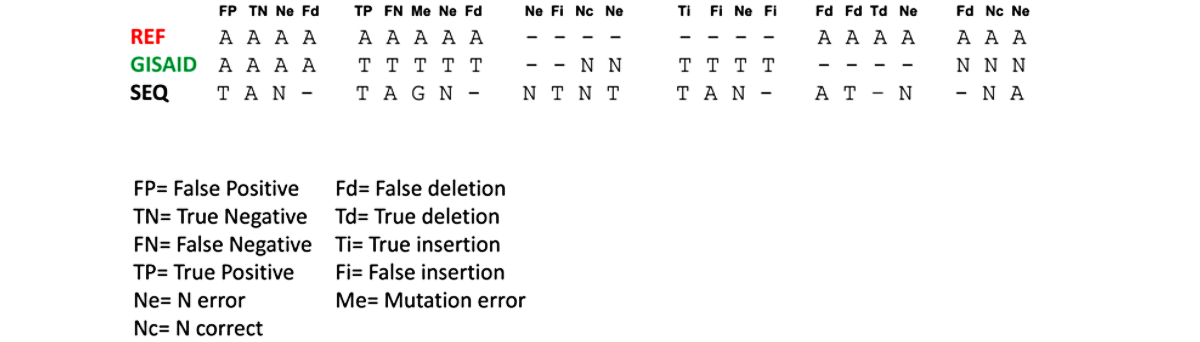
Classification scheme for genome assemblers, in which assembled genome sequences (SEQ) were compared with the corresponding submitted sequences (on GISAID) and with reference genome sequence “Wuhan-Hu-1” (REF). Nucleotide threesomes were classified using the following 11 categories: false deletion (Fd), false insertion (Fi), false negative (FN), false positive (FP), mutation error (Me), N correct (Nc), N error (Ne), true deletion (Td), true insertion (Ti), true negative (TN), true positive (TP).

The respective results were compared, aligning the sequences obtained using the 2 methods with the reference sequence Wuhan-Hu-1 (NCBI Acc. Numb. NC_045512.2), and the corrected sequence was submitted to GISAID, using MAFFT [20]. Then, each discordant position was evaluated following the classification reported in Figure 1. In particular, we evaluated true positives (TP; mutations correctly classified as real); false negatives (FN; mutations correctly classified as unreal); false positives (FP; mutations incorrectly classified as real); true negatives (TN; mutations correctly classified as unreal); corrected TN (positions unknown correctly classified as N); and TN error (positions unknown, incorrectly classified as N).

To test the performances with respect to mean coverage, linear regression correlation analysis was carried out for mean coverage and specific measures of accuracy.

## Results

In the computational evaluation, ESCA software was compared with the most often used assemblers for SARS-CoV-2 genome analysis on 228 SARS-CoV-2–positive samples.

**Figure 2:**
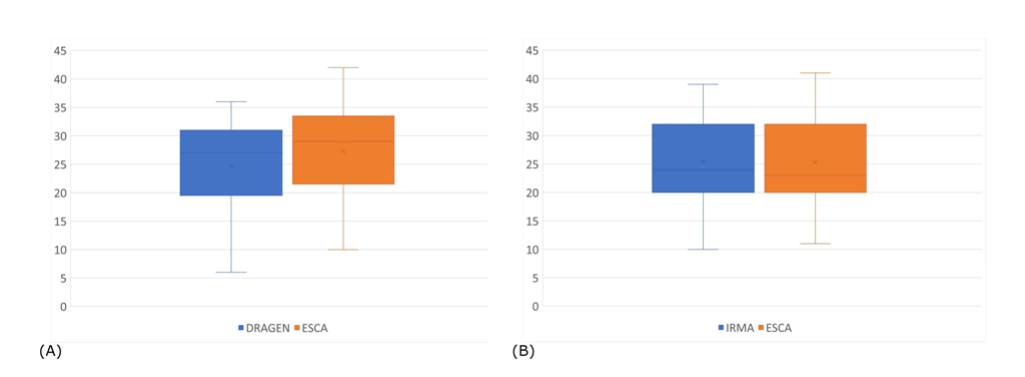
Comparison of true positive mutations between our Easy-to-use SARS-CoV-2 Assembler (ESCA) and the (A) Illumina DRAGEN tool and (B) iterative refinement meta-assembler (IRMA) recommended by Ion Torrent.

Sequencing was performed on Illumina MiSeq for 65 libraries, obtaining a median of 1.50 x 106 paired-end reads per sample (range: 0.02 x 106 to 4.56 x 106), and on Ion Gene Studio S5 Sequencer for 163 libraries, obtaining a median of 0.61 x 106 single-end reads per sample (range: 0.02 x 106 to 3.02 x 106). Using the ESCA reconstruction, the coverage point by point was calculated, and we observed that, in the Illumina sample, the point coverage was not uniform, although the mean coverage was quite high in all samples (average 3508X; range: 70-10,733). This could introduce error in genome reconstruction using some software. In this context, ESCA could reduce the error in regions with low coverage. In parallel, mean coverage obtained with Ion Torrent was 4966X (range: 94-19,917), but a higher uniformity was observed. The comparison of the coverage distribution is shown in Figure 2.

To evaluate the ESCA and DRAGEN/IRMA results, assembled genomes, the reference Wuhan-Hu-1, and the corrected genome of GISAID (Accession IDs available in Multimedia Appendix 1) were aligned with MAFFT [20].

At each position along the SARS-CoV-2 genome, the 24 available nucleotide combinations were classified in 11 mutation categories (Figure 1). For all sequences, the number of occurrences of mutation categories for each assembly software was then evaluated.

### Illumina Data

The comparison of ESCA with DRAGEN showed that, as expected, the mean number of mutations in genomes was very low (in the mean 28 position) and ESCA could correctly identify a mean 27 of 28 mutations (Figure 2A). Moreover, no FN positions were identified by ESCA. This is due to the pipeline design that reduces the error of introducing N where the coverage is not sufficient. The DRAGEN genome, instead, showed a mean of 25 of 28 TP and 3 FN positions. The absence of a mutation in specific positions could be essential to assigning the lineage, and the presence of FNs could modify the identification of the variants.

On the other hand, both ESCA and DRAGEN did not introduce FN, identifying 29,308 and 28,027 TN positions, respectively.

These results show an accuracy of 100% for ESCA and 99.99% for DRAGEN. Moreover, the sensitivity of ESCA compared with DRAGEN was 96.43% for ESCA and 89.29% for DRAGEN, and the specificity with both methods was 100%.

### Ion Torrent Data

Parallel to the previous comparison, ESCA compared with IRMA showed that both methods identified a mean 25 of 26 TP positions (Figure 2B) but did not induce FN. However, IRMA introduced a certain number of errors. In fact, the FP was 20 for IRMA, compared with 0 for ESCA. Once again, the introduction of mutations could induce error in the lineage assignation.

The accuracy of IRMA was calculated to be 99.93%, while it was 100% for ESCA.

Moreover, although the sensitivity was identical with the 2 methods (96.15%), the specificity was 99.93% for IRMA and 100% for ESCA.

### Performance Test

To evaluate the performance of each of the methods, linear regression correlation analysis was carried out with respect to mean coverage (Multimedia Appendix 2).

For IonTorrent single-end sequencing data, a significant positive correlation was found comparing coverage and TN for both IRMA and ESCA (r>0.15, *P*<.05), while for Illumina pair-end sequencing data, such a correlation was found only for DRAGEN (r>0.40, *P*<.05). This difference could be caused by a different error rate for the 2 sequencing techniques. These data suggest that all assembly methods are comparable in the case of high coverage samples, while ESCA seems to perform better for low coverage data.

## Discussion

### Principal Findings

The importance of rapidly obtaining and sharing high-quality whole genomes of SARS-CoV-2 is increasing with the emerging variant strains [14]. For this reason, the use of NGS custom amplicon panels can be a rapid and performant method for identifying viral variants. However, a lack of bioinformatic skills could be a problem in handling NGS raw data. Our pipeline ESCA provides help to laboratories with low bioinformatic capacity using a single command. Both of the more common methods for the analysis of Ion Torrent and Illumina data (IRMA and DRAGEN, respectively) have shown a certain amount of error that could induce false identification in variant assignation. On the contrary, the SARS-CoV-2 genome obtained by ESCA shows a reduced number of false insertions and false mutations and a higher number of real mutations.

### Limitations

This pipeline should be tested on a larger number of sequences and with other sequencing technologies.

### Conclusions

ESCA automatically produces a variant table output file, fundamental for rapidly recognizing variants of interest.

These results show how ESCA could be a useful method for obtaining a rapid, complete, and correct analysis even with minimal skill in bioinformatics.

## Appendix

Multimedia Appendix 1 Comparison of Easy-to-use SARS-CoV-2 Assembler (ESCA) and (first tab: Ion Torrent) iterative refinement meta-assembler (IRMA) and (second tab: Illumina) DRAGEN results for every assembled SARS-CoV-2 genome. DELerror: deletion unknown incorrectly identified; errorMut: number of incorrected mutations described; FN: false negative; FP: false positive; INScorr: insertion unknown correctly identified; INSerror: insertion unknown incorrectly identified; NCorr: positions unknown correctly classified as N; Nerror: position unknown incorrectly classified as N; TN: true negative; TP: true positive.

Multimedia Appendix 2 Pairwise linear regression correlation analysis (shown with the *P* value) for every parameter: DELerror: deletion unknown incorrectly identified; FN: false negative; FP: false positive; INScorr: insertion unknown correctly identified; INSerror: insertion unknown incorrectly identified; NCorr: positions unknown correctly classified as N; Nerror: position unknown incorrectly classified as N; TN: true negative; TP: true positive.

## Abbreviations

ACE2: angiotensin-converting enzyme 2
CDC: Centers for Disease Control and Prevention
ESCA: Easy-to-use SARS-CoV-2 Assembler
FN: false negative
FP: false positive
IRMA: iterative refinement meta-assembler
NGS: next-generation sequencing
TN: true negative
TP: true positive
